# Refractory Behavior in Plant Cells—Calcium Signaling Induced by Biotic Stress

**DOI:** 10.3390/plants15091395

**Published:** 2026-05-02

**Authors:** Mareike Kristin Keßler, Viktoria Fulek, Karsten Niehaus, Petra Lutter

**Affiliations:** 1 PlasmidFactory GmbH, Meisenstr. 96, D-33607 Bielefeld, Germany; mareike.kessler@plasmidfactory.com; 2 Center for Biotechnology—CeBiTec & Faculty of Biology, Bielefeld University, Universitätsstr. 25, D-33615 Bielefeld, Germany; kniehaus@cebitec.uni-bielefeld.de; 3 Medical School OWL, Bielefeld University, Universitätsstr. 25, D-33615 Bielefeld, Germany; viktoria.fulek@uni-bielefeld.de

**Keywords:** refractory behavior, calcium signature, plant cells, biotic stress, plant defense mechanism, elicitors and receptors

## Abstract

When in contact with microbes or other pathogens plants develop an induced defense response. This reaction is triggered by pathogen-derived molecules that provoke the so-called microbe-associated molecular pattern (MAMP)-triggered immunity (MTI) or pathogen-associated molecular pattern (PAMP)-triggered immunity (PTI). Recognition of a MAMP or PAMP by a pattern recognition receptor (PRR) activates rapid downstream signaling, manifested in, e.g., a rise in the cytosolic Ca^2+^ concentration. As a consequence, defense-related genes are expressed and antimicrobial substances are produced. There is also evidence that Ca^2+^-induced responses show a refractory behavior in plant cells, as the reaction to an identical stimulus applied shortly after the first one is strongly suppressed, if it can be observed at all. Subsequent elicitations over a longer period of time, on the other hand, can trigger stronger Ca^2+^ responses, which lead to so-called “defense priming”. Although refractory behavior has been documented in various plant cell types, its underlying function and causative mechanisms remain unclear. In this review article we give an overview of the refractory machinery, including elicitors, receptors, typical Ca^2+^ responses, and signal transduction pathways. We shed light on possible explanatory scenarios and address open questions.

## 1. Introduction

Plants lack a system of internal mobile cells and therefore did not evolve specialized mobile immune cells, as in the case of mammals. However, they still need to deal with biotic and abiotic stresses. Abiotic stress manifests itself through changes in temperature, salt concentration, lack of water, or mechanical disturbances. Plant cells also need to be capable of deploying various innate immune responses to ward off biotic stresses such as pathogens or toxins produced by pathogens [1]. Primarily, plants deploy non-host resistance, which describes a resistance mechanism against pathogens common to all plants of an entire species. The preformed barriers, such as waxy cuticles or cork cells, and antimicrobial secondary metabolites (e.g., phytoalexins) repel attempts by a pathogen to colonize the plant [2,3,4]. When in contact with microbes or other pathogens plants can develop an induced disease resistance: a microbe-associated molecular pattern (MAMP)-triggered immunity (MTI) or a pathogen-associated molecular pattern (PAMP)-triggered immunity (PTI). Recognition of MAMP or PAMP by pattern recognition receptors (PRRs) activates downstream signaling and leads to the expression of defense related genes and the production of antimicrobial substances [1,5,6,7]. Typical defense mechanisms are the production of reactive oxygen species (ROS), e.g., H_2_O_2_, or the biosynthesis of phytoalexins as well as the lignification of cell walls [8]. Defense gene expression is also activated by biotic stress. Part of the induced signal transduction involves mitogen-activated protein kinases (MAPK) which lead to the phosphorylation of WRKY transcription factors (TFs), which in turn bind to the DNA-binding domain containing the WRKYGQK motif, regulating defense gene expression [9]. An overview of the roles of WRKY TFs in the context of stress responses in plants and possible agricultural applications is given by Wani et al. [10]. The induction of an oxidative burst can be observed minutes after pathogen contact, while the induction of PAMP-induced gene- as protein-expressionand the synthesis of defense-related small molecules might take hours.

An exposure of living plant cells to an elicitor (stimulus) often influences the response to a later stimulus, which is known as defense priming or trained immunity or acquired immunity [1,11]. Long-term plant cell memory can be obtained through the modification of histones [12,13], demethylation of DNA in the promoter region of defense genes [14,15], the deposition of dormant cellular signaling enzymes, as, e.g., MAPK [16,17], or an increased level of PRRs [18,19]. In contrast to the trained immunity, plants also exhibit a short-term adaptation. The refractory behavior, acting on a far smaller timescale, is observed in many plants that are exposed to a stress situation. In this case, the reaction becomes progressively smaller upon repeated stimulation and a refractory period is required before a full response is observed again [20].

In this review, we provide an overview of typical elicitors as well as their receptors that are considered to trigger biotic stress responses in plants. We place particular emphasis on calcium signaling in response to an elicitor stimulus, which leads to the activation of defense mechanisms. As the refractory behavior of the calcium signature has not been thoroughly discussed in the literature yet, we provide a view on current and future research approaches.

## 2. Elicitors and Receptors

Elicitors (PAMPs) of a plant defense response are molecules mostly produced or released by pathogens. They mainly consist of oligosaccharides, peptides, lipids or proteins. These molecules can be recognized by cell surface-localized receptors and activate plant immunity [21]. Plant immunity is expressed in the rapidly induced production of reactive oxygen species (ROS), which have antimicrobial effects and crosslink cell wall compounds, as well as in programmed cell death and the activation of resistance and defense genes [22,23,24]. However, not only biotic molecules can induce plant immunity; abiotic factors, such as temperature and mechanical pressure, can elicit plant immunity as well [25,26,27,28].

In general, elicitors can be identified as conserved structural cell components that are part of the cell wall or cytoplasm of the pathogen like glucan, chitin, flagellin and lipopolysaccharides (LPSs), or even released by pathogen derived enzymes from the host cell, as, e.g., oligogalacturonides (OGs) [29]. Oligosaccharides released by plant enzymes from the fungal cell wall induce, e.g., multiple defense reactions and resistance responses like, e.g., oxidative burst through H_2_O_2_ production in grapevine cells [30,31]. Pep-13, an oligopeptide fragment of a 42 kD *P. sojae* cell wall glycoprotein, leads to an induced Ca^2+^ response in parsley cells and activates production of ROS and phytoalexins [32]. Cytosolic Ca^2+^ signals in *N. tabacum* cell cultures can also be induced by elicitors such as penta-N-acetylchitopentaose (Ch5) oligosaccharides [33], among others. Calcium signaling is not limited to pathogenic plant–microbe interactions. During the symbiosis between legumes and rhizobial bacteria, specific nodulation signals known as Nod factors are secreted. Nod factors consist of a chitin oligomer backbone with an attached fatty acid. These factors can induce a cytosolic Ca^2+^ signal, which has been measured in aequorin-expressing soybean cells [34,35].

The perception of extracellular elicitors involves surface-based receptors that will interact with patterns derived from the pathogen. The PRR that has bound an elicitor triggers a stimulus in the cytoplasm, which in turn triggers different defense mechanisms. However, only a few receptors that interact with elicitor molecules have been identified [29,36]. A prominent example is the Flagellin-sensitive 2 (FLS2) receptor, which was identified by Gomez-Gomez and Boller in 2000 [37]. It is a PRR with an extracellular leucine-rich repeat motif (LRR), a transmembrane domain, and a cytoplasmatic serine/threonine kinase domain. Plants employ PRRs for sensitive and rapid detection of the potential danger caused by microbes and other pathogens. In *Arabidopsis*, perception of flagellin occurs by recognition of the most conserved part represented by the peptide flg22. Perception of flg22 by FLS2 activates a downstream MAPK pathway [38]. For chitin-triggered immune responses, receptors with extracellular lysin motif (LysM) domains were found to be essential [39]. Johnson et al. introduced the novel elicitor cellotriose, a compound isolated from the cell wall extract of the fungus *Piriformospora indica*, that induces a mild defense-like response in host plant roots, which in turn leads to resistance to biotic and abiotic stress [40,41]. Degradation of the plant cell wall also leads to cellotriose, which is recognized as a damage-associated molecular pattern (DAMP) via the cellooligomer receptorkinase 1 (CORK 1) in *A. thalina* roots [42]. Bacterial lipopolysaccharides are of special interest, as these molecules are perceived by animal cells with outstanding sensitivity. In 2011 it was shown that the LPS of a plant pathogen is sensed by a non-host *N. tabacum* cell culture, resulting in a characteristic calcium signature and oxidative burst [43]. Further work showed that the lipid A subunit of the molecule is recognized by the plant [44] and that the LPS is internalized via receptor-mediated endocytosis [45]. As for other PAMPs, a specific receptor kinase, here the bulb-type lectin S-domain-1 receptor-like kinase LORE (SD1-29), seems to recognize the LPS [46]. Recent analysis in *A. thaliana* mutants indicates that also other receptor kinases are involved [47]. In crop plants such as *Glycine max*, pathogen recognition receptors (PRRs) constitute a large and diverse receptor repertoire at the plasma membrane. Although only a few PRRs have been experimentally characterized, genomic analyses indicate that soybean encodes more than a hundred receptor-like kinases and receptor-like proteins with potential PRR functions [48]. Further work will be needed to identify the ligands of these receptors and functionally group the connected signal transduction networks.

In Table 1 we provide an overview of the most common PAMPs with their known receptors. A more detailed summary of elicitors and receptors can be looked up in the existing literature [22,29,49].

## 3. Calcium Response to Elicitation and Refractory Behavior

Calcium (Ca^2+^) is an essential plant nutrient and intracellular second messenger [66], which enters the cytoplasm of the cell either through permeable ion channels in the plasma membrane or from internal stores. A high Ca^2+^ concentration in the cytosol is toxic, and Ca^2+^ homeostasis is important [67]. Therefore, Ca^2+^—ATPase and H^+^/Ca^2+^—antiporters remove cytosolic calcium and relocate it in the apoplast or intracellular organelles that act as Ca^2+^ stores, such as the vacuole, the endoplasmatic reticulum (ER), chloroplasts and mitochondria [68,69]. The removal of cytosolic Ca^2+^ is essential for plant growth; a change in cytosolic Ca^2+^ levels addresses the switch between plant growth and immunity [70].

Selective calcium channels are activated by cytosolic signal molecules, such as inositol trisphosphate (IP_3_), inositol hexakisphoshate (IP_6_) or cyclic guanosine monophosphate (cGMP) [71]. These second messengers are involved in calcium-mediated signal transduction and target ligand-gated Ca^2+^ channels within organelle membranes to release calcium into the cytosol [72]. There is evidence of cross-talk between Ca^2+^ and cyclic nucleotides to selectively regulate the activity of signaling pathways [73]. In their mini-review Park and Shin give a summary of various ion channels involved in Ca^2+^ influx (e.g., cation channels, voltage-dependent channels, two-pore channels) as well as membrane proteins to regulate Ca^2+^ efflux (e.g., Ca^2+^-ATPase and Ca^2+^/H^+^-exchangers) [74].

A diverse range of stresses can initiate a cellular response that is characterized by a rapid influx of Ca^2+^ from the organelles that serve as internal stores or from the apoplast. As a consequence, the Ca^2+^ concentration in the immediate environment is thoroughly increased [72]. Calcium dependent processes take place in the cytoplasm, mitochondria [75], chloroplasts [76] and nuclei [77,78].

A rise in the cytosolic Ca^2+^ concentration in plant cells, usually effected by an influx from the apoplast and organelles, is a typical response to biotic and abiotic stresses. These perturbations in cytosolic calcium levels are often referred to as Ca^2+^ signature. In addition to the influx and efflux of Ca^2+^ from different internal stores, which may shape the cytosolic Ca^2+^ variations, calcium influx through plasma membrane channels has been shown to be required [79]. The IP_3_ release is Ca^2+^ dependent [80]. As for intracellular Ca^2+^ release channels, inositol 1,4,5-trisphosphate receptors (IP_3_R) are the most widely expressed ones [71,81].

The Ca^2+^ signature is embedded in a fast and interconnected signaling network that also involves other ion fluxes (e.g., potassium), protein phosphorylation, and the generation of additional second messengers. These transient Ca^2+^ dynamics function as part of a molecular decision-making system that determines the cellular response to external stimuli [82]. The Ca^2+^ signature elicited by a given stimulus is characterized by its amplitude, duration, frequency, and subcellular location. This signature encodes specific information that, upon decoding by downstream components, contributes to the generation of defined physiological outputs [83]. As in the signal transduction of animal cells, it seems that also in plant cells the calcium signature observed upon elicitor perception is only part of a more complex signal transduction network. The calcium transient is a requirement, but is not sufficient on its own to induce the whole set of defense reactions. A sustained high cytosolic Ca^2+^ concentration, however, is implicated in apoptosis. In order to effect adaptive responses, the cytosolic Ca^2+^ perturbations must either be of a comparably low amplitude, if sustained over a longer time period, or take a transient form [20,84]. The specific kinetics of this calcium response are influenced by many factors, e.g., channel density and state, intracellular store replenishment, and other second messengers. Moreover, different signals will lead to calcium signatures of different shapes, and the induced kinetics are unique. Some cellular states will enable calcium oscillations [85]. Changes in calcium concentration not only occur in response to biotic or abiotic stress, but oscillations in the calcium concentration associated with the nucleus are at the core of the symbiosis signaling pathway. Nod factors, released from rhizobia, bind to their receptors on legumes. NFR1 and NFR5 together form a heterodimer and the transduced signal leads to calcium oscillations in the nucleus, where genes that are important for the establishment of symbiosis are activated [86,87].

**Figure 1:**
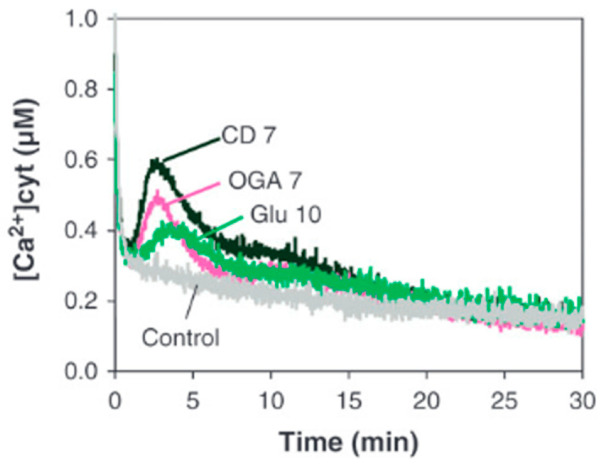
Change in cytosolic Ca^2+^ concentration in aequorin-transformed grapevine cells after treatment with β-1,4 cellodextrins (CD 7, 0.5 mg/mL), β-1,3 glucans (Glu 10, 0.5 mg/mL) or α-1,4 oligogalacturonides (OGA 7, 0.5 mg/mL). The same volume of water was used as a control. Figure modified from [31].

In contrast to symbiosis-associated calcium oscillations, a calcium response to biotic stress typically occurs in the cytoplasm. When grapevine cells were challenged with cellodextrins (CD), a rapid and transient increase in the cytosolic Ca^2+^ concentration was observed [31]. Figure 1 shows a typical Ca^2+^ signature, which is characterized by a steep rise in the Ca^2+^ concentration, followed by a comparably weaker decline, right after the peak has been reached.

Although the cytosolic Ca^2+^ concentration depends on the deployed elicitor concentration, the Ca^2+^ response becomes saturated once a specific elicitor concentration is reached. Elicitation of, e.g., apoaequorin-transformed parsley cells with Pep13, induces a Ca^2+^ response that already becomes saturated at a concentration >5 nM [32]. Obviously, the plant detection system for PAMPs is highly sensitive. Moreover, it shows a very short response time, which is typically below one minute (see also Figure 1; [32]).

After the treatment of transgenic soybean cells with OGs, the cytosolic Ca^2+^ response also appears to be a rapid and transient event. The degree of polymerization of the OGs has a strong influence on the kinetics of the Ca^2+^ responses, a higher degree of polymerization results in a higher cytosolic calcium concentration [64]. Dose-dependent treatments with cryptogein (Cry) and OGs were performed in aequorin-transformed *N. tabacum* cell suspension cultures, inducing a biphasic cytosolic Ca^2+^ rise, which increases with higher elicitor concentration. Compared to the Cry-induced Ca^2+^ elevation, *N. tabacum* cells treated with OGs showed a faster calcium reaction and a faster return to the basal calcium level [79].

**Figure 2:**
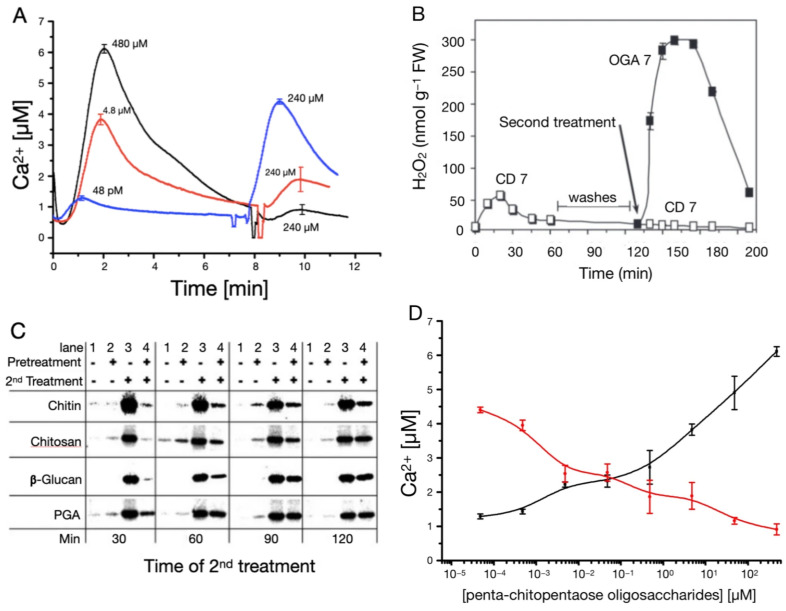
Refractory behavior of Ca^2+^ signaling measured in terms of cytosolic Ca^2+^ concentration, H_2_O_2_ production and MAPK activity. (**A**) Tobacco cell cultures were elicited with 480 μM, 4.8 μM and 48 pM of Ch5. Approximately 7 min later, a second elicitation with a Ch5 stimulus of 240 μM was carried out. (**B**) H_2_O_2_ production in grapevine cell suspensions after successive addition of CD 7 and OGA 7, demonstrating refractory behavior. Cells were first treated at time 0 with 0.5 mg/mL CD 7, washed three times with fresh medium within the second hour after first treatment, then treated a second time with 0.5 mg/mL CD 7 (open squares) or with 0.5 mg/mL OGA 7 (black squares), respectively. (**C**) MAPK activity in reaction to successive oligosaccharide elicitor (OE) treatments. *L. peruvianum* suspension-cultured cells were pretreated with the OEs chitin (100 µM chitotetraose), chitosan (1.7 µg/mL), ß-glucan (10 µg/mL), or polygalacturonic acid (PGA, 830 µg/mL), or were left untreated. At 30, 60, 90, and 120 min thereafter, treated and untreated cells were either left untreated or were exposed to the same OE a second time. (**D**) Dose–response diagram for first (black curve) and second (red curve) elicitations. Ch5 concentration of the first elicitation was increased, while the second elicitation was constantly held at 240 µM. Ca^2+^ responses were set in relation to stimulus level of first stimulation. Modified from [31] (**B**), ref. [33] (**A**,**D**), ref. [90] (**C**).

Already in the early 1990s there was evidence of refractory behavior regarding Ca^2+^ responses, as tomato cells elicited with chitin fragments did not respond to a second stimulus applied shortly after the first one [88]. Obviously, there is a period of time—the refractory period—after a stimulus in which the cells can only respond less or not at all to a stimulus of the same kind. However, throughout this refractory period the tomato cells remain responsive to a different stimulus [58,88]. Even if the elicitor is “washed out” between two elicitations, the cells remain less responsive to the second stimulation. As an example, grapevine cells pretreated with CD (CD 7 at 0.5 mg/mL) did not respond to a second treatment with CD 7 (Figure 2B). However, they responded to an application of a different elicitor type, e.g., OG (OGA 7 at 0.5 mg/mL) [31]. Other oligosaccharide elicitors (OEs: chitosan, chitotetraose, ß-glucan and PGA) were shown to induce a refractory state as well. Given that the receptor type is an essential element of stimulus reception, whereby different stimuli might in rare cases also be received by a common receptor [89], we can speculate that the receptor itself is involved in the refractory behavior of this system.

Refractory behavior can also be observed at the MAPK kinase activity level. MAP kinases can be activated by a Ca^2+^ response, and their activity reaches its maximum after ten minutes. Once active, further responses are also reduced within a specific time interval. MAPK activity levels obviously recover after 120 min, as can be seen in Figure 2C. After two hours the response to a second treatment is almost as strong as the one induced without pretreatment [90]. Downstream, MAPK cascades act as temporal integrators, preferentially responding to signals of defined duration or persistence and translating transient early events into stable transcriptional reprogramming.

Small GTPases—particularly plant Rho-of-plants (ROP/RAC) proteins—act as central molecular switches that couple stimulus perception with activation, spatial organization, and amplification of the Ca^2+^-ROS-MAPK network. Rather than functioning as linear intermediates, they provide regulatory control over signal initiation, localization, and intensity. In addition to ROS regulation, small GTPases also influence Ca^2+^ dynamics itself. By affecting membrane organization, cytoskeletal architecture, and possibly channel positioning, ROPs modulate the spatial patterning of Ca^2+^ influx and activation of the NADPH-oxidase, which is responsible for the production of ROS, which will influence pathogen as symbiont interactions [91]. This is particularly evident in processes such as tip growth, where localized ROP activity establishes Ca^2+^ gradients. In immune signaling, similar principles likely apply at the scale of plasma membrane nanodomains, enabling spatially confined ROS and Ca^2+^ microdomains that shape downstream decoding. More research is needed to integrate the role of plant GTPases and their respective regulatory proteins in the Ca^2+^-ROS-MAPK network.

On the other hand, it is possible to trigger an increased second response if the level of the first elicitation is sufficiently reduced. The corresponding Ca^2+^ responses of tobacco cell cultures treated with Ch5 show this behavior in more detail (Figure 2A, ref. [33]). A first elicitation with 48 pM, followed by a second elicitation with 240 µM, leads to a Ca^2+^ response even bigger than the response to a single stimulation with, e.g., 4.8 µM. When looking at the dose–response curves of the first and second elicitation separately, the first elicitation shows the typical distinct rise, while the second elicitation shows the expected decrease (Figure 2D, ref. [33]). Generally, Figure 2 gives an overview of the refractory behavior of Ca^2+^ responses, measured in terms of Ca^2+^ concentration, H_2_O_2_ (Figure 2B, ref. [31]) production and MAPK (Figure 2C, ref. [90]) activity. In this context it is noteworthy that all tested PAMP receptors induce MAPK activity (see Figure 2C) through Ca^2+^ signaling, but show a differential activation of the various MAPK pathways [92].

LPS and cyclic adenosine monophosphate (cAMP) induce a cytosolic Ca^2+^ elevation in intact leaves of wild type *Arabidopsis thaliana*, but are absent in leaves lacking cyclic nucleotide-gated cation channels [93]. The plasma membrane cation binding protein 1 (PCaP1) is required for late defense responses to elicitors and for the recovery of full responsiveness to OGs after an elicitor treatment [94].

**Figure 3:**
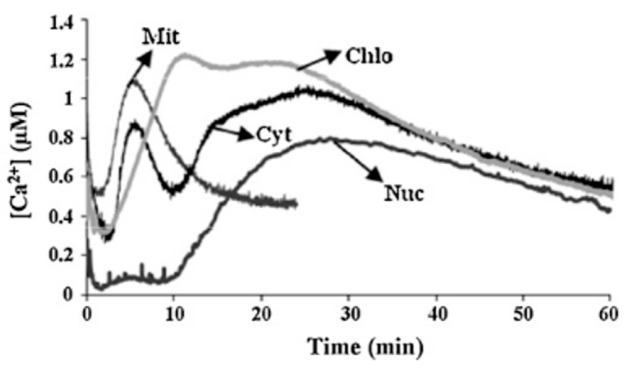
Ca^2+^ responses induced by Cry (100 nM) in the cytosol (Cyt), chloroplasts (Chlo), mitochondria (Mit) and nucleus (Nuc). Figure modified from [79].

As already stated earlier, the Ca^2+^ increase in the cytosol is affected by the Ca^2+^ influx from the apoplast or from other intracellular organelles [20]. But Ca^2+^ signatures are not exclusively observed in the cytosol. Ca^2+^ is typically stored in the vacuoles, the ER, the mitochondria and the chloroplasts, and Ca^2+^ signatures are observed in the cytosol, the chloroplasts, the mitochondria, and the nucleus (Figure 3). Apart from the above-mentioned dose-dependent treatments, the two PAMPs (Cry and OGs) were used to study whether Ca^2+^ variations could affect important physiological processes in organelles, such as photosynthesis and mitochondrial respiration. Cry, through Ca^2+^ signaling, causes perturbations in two important organelle functions, namely mitochondrial respiration and chloroplastic energy dissipation. This strengthened the idea that Ca^2+^ in organelles contributes to plant defense signaling [79].

## 4. Possible Causes of Refractory Behavior in the Context of Calcium Signaling

A rise in the cytosolic Ca^2+^ concentration, the so called “Ca^2+^ answer”, in response to biotic or abiotic stresses, leads to the expression of defense-related genes, ROS production or apoptosis. However, little is known about what triggers the refractory behavior in the context of Ca^2+^ responses.

**Figure 4:**
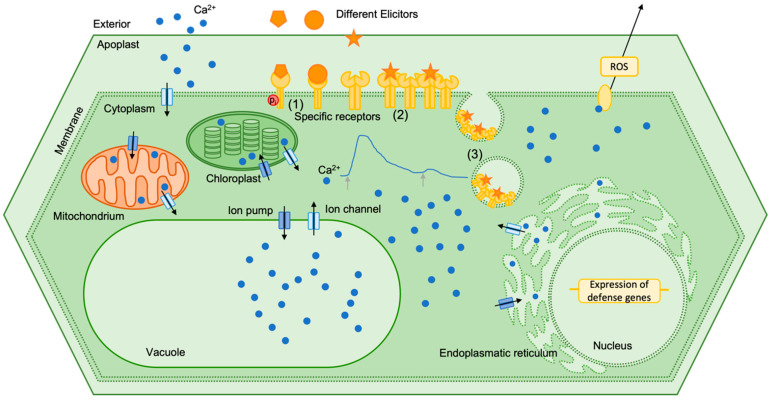
Model for refractory behavior in calcium signaling. Different PAMPs acting as elicitors are detected by surface-located PRRs. Perception of the signal leads to an influx of calcium ions through different calcium channels, resulting in an induced cytosolic calcium concentration. Thus, different defense responses are triggered, like, e.g., ROS production or expression of defense-related genes. After the event calcium is removed from the cytosol via specific calcium pumps. Numbers indicate different possibilities that are responsible for refractory behavior: (1) phosphorylation of PRRs leads to conformational changes and disables interaction with elicitor molecules, (2) clustering of receptors reduces the possible binding sites and reduces interaction between PRR and PAMP molecules, and (3) receptor endocytosis removes the PPR with bound elicitor molecules from the cell surface; the plant cell cannot respond to a new stimulus until new PRRs are synthesized and brought to the cell surface.

Cytosolic calcium signals are the consequence of a complex perception system that releases calcium from external as well as internal stores. And signal transduction of the cytosolic calcium transient is not less complex. Figure 4 illustrates the journey of the calcium ions to show possible causes of refractory behavior.

Phosphorylation of specific PRRs is one possible causes of refractory behavior [95]. The conformational change can prevent the connection to an elicitor. It could be shown that MAPK are also reversibly phosphorylated and may undergo a refractory state before the next activation [90]. Interestingly, in most studies phosphorylation leads to the activation of PRRs and precedes all responses and intracellular signaling events. Leucine-rich repeat receptor kinases (LRR-RK) located at the plasma membrane in particular play an important role in the perception of extracellular signals. For FLS2 (flagellin-sensitive 2) the interaction with BAK1 (BRI1-associated receptor kinase 1) was shown to be required for signal transduction. After stimulation with flg22 the phosphorylation of the FLS2-BAK1 complex stimulates signal transduction and leads to activated plant defense reactions [96]. Tyrosine phosphorylation in particular plays an important role in the activation of PRRs. The tyrosine kinase inhibitor A23 reduced BIK1 (botrytis-induced kinase 1) phosphorylation and the production of ROS, suggesting that tyrosine phosphorylation regulates immune signaling induced by PAMPs [97]. Not only can PRRs be phosphorylated, but also the phosphorylation of Ca^2+^ channels could be a possible cause for refractory behavior. Receptor-like cytoplasmatic kinases are able to phosphorylate ion channels to regulate ion transport and early signaling during plant-triggered immunity [98]. The loss of the cyclic nucleotide-gated calcium channels leads to a hypersensitive temperature-dependent Ca^2+^ influx and a corresponding hyper-thermoresponsive profile of heat shock response activation [99]. In addition to phosphorylation, other modifications of receptors, channels and downstream kinases are also involved. Ubiquitiniation, lipidation, acetylation and SUMOylation are reported protein modifications that can contribute to the refractory behavior of plant cells in response to stress [95].

Another cause for refractory behavior could be receptor clustering. The hypothesis is that receptors somehow interact, so that within a stimulated cluster there are also receptors that are not bound to an elicitor molecule, thereby reducing the number of free receptors for a second stimulus. As an example, the quorum-sensing molecule diffusible signal factor (DSF) impaired the FLS2 nanoclustering on the cell surface, which therefore desensitized *Arabidopsis*’ immune response to flg22 [100].

A most likely cause for refractory behavior is receptor endocytosis. After the internalization of the receptors an additional elicitation signal cannot be transmitted until new receptors are synthesized. Endocytosis is a well-known mechanism for cellular desensitization, where ligand-bound receptors from the cell surface are removed. In many plant defense studies endocytic processes could be observed. Shortly after elicitation with a labeled elicitor fluorescence in vesicle-like structures is found [45,101,102,103]. A possible role in endocytosis after elicitation is played by PCaP1. PCaP1 is organized in membrane microdomains and is internalized in endocytic vesicles in response to OGs. However, endocytosis is not altered in mutants lacking PCaP1 [94].

## 5. Refractory Behavior and Defense Priming

There is considerable evidence that cytosolic Ca^2+^ signatures can be modified by previous exposure to a stimulus. The magnitude of the Ca^2+^ elevation becomes progressively smaller upon repeated stimulation, and a refractory period of several minutes or even hours is required before a full response is observed again. There is also evidence that the Ca^2+^ signatures elicited by one environmental challenge can be modified by prior exposure to a contrasting one [84,104]. When primed, plants respond to very low stimulation with a faster and stronger defense than unprimed plants [105].

This suggests that plants can discriminate between a single stress and repeated stresses and can modify their expression of the stress-responsive genes [106]. In Table 2 an overview of responses to biotic and abiotic stresses is shown. Changes in enzyme activity, cytosolic Ca^2+^ concentration and in metabolite levels may imply defense priming and memory to pathogen contact. By becoming more resistant to harmful stimuli on the one hand and less sensitive to harmless stimuli on the other, plants develop a so-called learning memory [107]. Transcriptional regulation enables plants to adapt to different stress environments [10]. To gain a better understanding of stress memory and priming in plants, changes at the epigenetic, transcriptional, proteomic and physiological level have to be considered [108].

Pathogen-induced Ca^2+^ signaling is intimately linked to the generation of ROS, underscoring the evolutionary conservation of this defense module. In plants, the NADPH oxidase RbohD functions as a central ROS-producing enzyme, and its activation is at least partly mediated by Ca^2+^. Notably, both the structural organization of NADPH oxidases and their regulation by Ca^2+^ are conserved across plants and animals [109], highlighting the ancient origin of this signaling architecture. Consequently, mechanistic insights gained from model species such as *Arabidopsis thaliana* and *Nicotiana tabacum* are frequently transferable to agronomically important crop plants [110].

Comparative transcriptome analysis in *Brassica napus* has provided further support for this concept. In a study contrasting the low aphid-susceptible cultivar “APL01” with the highly susceptible cultivar “Holly” under aphid challenge, genes associated with Ca^2+^ signaling were identified as differentially regulated in relation to resistance phenotypes [111]. These findings emphasize the functional relevance of Ca^2+^-dependent pathways in crop defense responses. For the identification of robust marker genes applicable to breeding programs, defense-associated signaling must be understood as an integrated network. In particular, calcium-dependent protein kinases (CDPKs) and MAPKs represent critical nodes linking Ca^2+^ dynamics to transcriptional reprogramming [112,113]. In addition to the search for a gene-marker-based selection of traits more resistant to stress, small molecules could enable the development of novel agrochemicals. Small molecules such as salicylic acid, jasmonate and other secondary metabolites are involved in signal transduction in plant decision making [114]. A recent study on rice showed the induced plant resilience to stress through the involvement of secondary plant metabolites and calcium signaling [115].

Mechanistically, the refractory phase arises from multilayered negative feedback, including Ca^2+^-dependent inactivation of Ca^2+^-permeable channels, receptor desensitization and turnover, depletion and refilling kinetics of intracellular Ca^2+^ stores, and induction of inhibitory signaling components such as phosphatases and Ca^2+^-binding regulators. Functionally, refractoriness prevents signal saturation and cytotoxic Ca^2+^ overload, but more importantly, it provides temporal resolution and encoding capacity to the Ca^2+^ signaling network.

The systematic identification of central signal transduction hubs within this network will provide a rational basis for breeding crop varieties that are able to respond in a timely and effective manner to pathogen attack.

## 6. Conclusions and Outlook

In summary, plant immunity is still a very complex phenomenon, although many defense mechanisms are known. Different elicitors, typically made up of molecules such as oligosaccharides, peptides, lipids, and proteins can trigger different plant defense mechanisms. Induced Ca^2+^ signatures are typically observed in the cytosol, the chloroplasts, the mitochondria, and the nucleus [116]. The calcium responses to biotic stimuli can have different meanings. Oscillating calcium responses are associated with symbiotic signaling pathways while transient calcium responses trigger defense-associated pathways [117]. At the beginning of the defense mechanism cascade a PRR binds to an elicitor. To date only a few receptors are known that interact with elicitor molecules [29]. Work in cell cultures is an excellent tool for analyzing basic mechanisms of Ca^2+^ signatures, but may fail to understand the reaction of the entire plant. Recent work by Wang et al. [118] shows the complex interplay of receptors, channels, and Ca^2+^ signatures at the tissue level.

In many studies the calcium responses of different model plants to elicitors are recorded. However, the reactions to a second elicitation with the same elicitor can be of a different type. A refractory period can be observed, i.e., a period of time after a stimulus in which the cells can only respond less or not at all to a stimulus of the same kind. However, during this refractory period cells remain responsive to a different stimulus. This refractory behavior is observed in many plants that are exposed to stress. Calcium levels can be reduced or vanish; however, sometimes it is possible to trigger even an increased second response, which is observed in the context of defense priming or trained or acquired immunity. There is considerable evidence that cytosolic Ca^2+^ signatures can be modified by previous exposure to a stimulus.

In search of the causes of refractory behavior, phosphorylation of specific PRRs is one possible option. Receptor clustering, which reduces the number of free receptors for a second stimulus, is another plausible cause. Receptor endocytosis is yet another probable reason for a refractory time interval. In addition, experiments with repeated elicitation can help us to find out more about the refractory interval period. Interestingly, Yakushiji et al. studied the influence of bacterial DNA on *Arabidopsis thaliana* and found that the endocytosis inhibitors wortmannin and amantadine significantly inhibited the plant’s defense response that had been DNA-induced [119].

In the context of the plant immune system, it is important to obtain a clear definition of “stress”. Biotic and abiotic stimuli can act as stressors and trigger defense reactions. The calcium response at the cellular level could be utilized to set a standard measure and make different forms of stress more comparable. In their review on Ca^2+^ signatures in response to environmental stresses [120] the authors discuss strategies to identify potential Ca^2+^ channels as stress sensors. In view of the growing number of confirmed calcium channels, Jiang and Ding raise the question of their spatio-temporal function [121].

As priming is a part of the induced resistance that neither significantly alters growth nor fruit or seed set, its importance increases in terms of disease control [122]. The thorough study of the plant immune system, including at the omics level, will support crop development for food safety and security. Changes in the epigenome, transcriptome, proteome and metabolome in reaction to stress can inscribe themselves in the memory of the plants, which enables them to respond to future stress in a more pronounced way [123]. Recent findings by Jia et al. [124] show that deciphering the link between NLR (leucine-rich repeat receptor)-mediated Ca^2+^ signaling and ROS transport is crucial in plant immunity. The group identified a calcium-dependent protein kinase regulating ROS signaling during effector-triggered immunity. In plants, the relationship between diverse environmental inputs and specific physiological outputs is generated through the dynamic properties of the interconnected Ca^2+^-ROS-MAPK signaling network rather than through strictly linear signaling pathways. Distinct stimuli such as elicitors, cold shock, or mechanical perturbation activate stimulus-specific receptors or mechanosensitive channels, thereby producing characteristic cytosolic Ca^2+^ signatures that differ in amplitude, duration, frequency, and spatial distribution. Signal transduction, or—in other words—information processing, is a property of all living cells. Calcium signals are part of a complex and multidimensional network that integrates numerous information to generate an optimal output for the particular cell, organ or individuum. A systems biology approach, including modeling of this network, is needed to better understand this decision-making network [82].

## Figures and Tables

**Table 1:** Overview of the most common pathogen-associated molecular patterns (PAMPs) with known receptors and biological responses. Modified from [22,49].

PAMP (and Pathogens)	Known Receptor Genes	Biological Response	Reference
Cellotriose (Fungi, *Piriformospora indica*)	Not identified (n.i)	production of ROS, changes in membrane potential, expression of genes involved in growth regulation and root development	[40]
Cerebrosides A,C (Fungi, *Magnaporthe* spp.)	n.i.	phytoalexin production in rice	[50]
Chitin/chitosan, Ch5 (Fungi)	CEBIP, CERK1 (LYK1, RLK1), LYM2, LYK4	perception of chitin oligosaccharide elicitor for defense responses	[39,51,52,53,54]
Cold shock protein (Gram-negative bacteria, Gram-positive bacteria)	Cold shock protein receptor (CORE)	oxidative burst	[55]
Cryptogein (Fungi, *Phytophthora cryptogea*)	n.i.	induces local and distal defense responses, elicits leaf necrosis	[56]
Elongation factor (EF-Tu) (Gram-negative bacteria)	EFR	determines the specific perception of EF-Tu	[57]
Ergosterol (Fungi)	n.i.	induces ion fluxes in tomato	[58]
Flagellin (flg22) (Gram-negative bacteria)	FLS2	flagellin-binding initiates the innate immune MAP kinase signaling cascade, resulting in enhanced resistance against pathogens; activates a downstream MAPK pathway	[37,38]
Harpin (HrpZ) (Gram-negative bacteria)	n.i.	HR-like cell death, induces defense responses in various plants	[59]
Invertase (Yeast)	n.i.	activation of the phenylpropanoid pathway, ethylene production in tomato	[60]
Lipid-transfer proteins (Elicitins) (Oomycetes *Phytophthora* spp., *Pythium* spp.)	n.i.	HR-like cell death, induces defense responses in tobacco, systemic acquired resistance to microbial infection	[61]
LPS (Gram-negative bacteria)	n.i.	oxidative burst	[43]
Necrosis-inducing proteins (Bacteria *Bacillus* spp., fungi *Fusarium* spp., oomycetes *Phytophthora* spp., *Pythium* spp.)	n.i.	HR-like cell death	[62]
OGs (Bacteria *E. carotovora*)	Potato receptor-like kinase (PRK1-4)	induce a rise in cytosolic calcium concentration	[63,64]
Pep-13 (Glycoprotein) (Oomycete *Phytophthora sojae*)	PEPR1, PEPR2	senses an endogenous elicitor that potentiates PAMP-inducible plant responses	[32,65]
TMV replicase (Protein of the Tobacco Mosaic Virus)	n.i.	senses the TMV replicase and induces rapid cell death in tobacco	[36]

**Table 2:** List of effects and outcomes in response to abiotic and biotic stresses. Modified from [106].

Trigger	Effect	Outcome
Abiotic stress (e.g., UV radiation, water, temperature, salinity)	Changes in DNA methylation/chromatin pattern	Improved stress management/memory
Biotic stress (PAMPs, MAMPs)	Accumulation of transcription factors	Forgetting (e.g., autophagy)
	Changing phytohormone/ metabolite levels	
	Post-translational modifications	

## Data Availability

No new data were created or analyzed in this study.
